# Legume Diversity Patterns in West Central Africa: Influence of Species Biology on Distribution Models

**DOI:** 10.1371/journal.pone.0041526

**Published:** 2012-07-23

**Authors:** Manuel de la Estrella, Rubén G. Mateo, Jan J. Wieringa, Barbara Mackinder, Jesús Muñoz

**Affiliations:** 1 Departamento de Botánica, Ecología y Fisiología Vegetal, Universidad de Córdoba, Córdoba, Spain; 2 Institut de recherche en biologie végétale and Département de Sciences biologiques, Université de Montréal, Montréal, Québec, Canada; 3 Real Jardín Botánico (RJB-CSIC), Madrid, Spain; 4 Institute of Botany, University of Liège, Liège, Belgium; 5 Netherlands Centre for Biodiversity Naturalis (section NHN), Herbarium Vadense (WAG), Wageningen University, Wageningen, The Netherlands; 6 Herbarium, Library, Art and Archives, Royal Botanic Gardens Kew, Surrey, United Kingdom; 7 Universidad Tecnológica Indoamérica, Ambato, Ecuador; New York State Museum, United States of America

## Abstract

**Objectives:**

Species Distribution Models (SDMs) are used to produce predictions of potential Leguminosae diversity in West Central Africa. Those predictions are evaluated subsequently using expert opinion. The established methodology of combining all SDMs is refined to assess species diversity within five defined vegetation types. Potential species diversity is thus predicted for each vegetation type respectively. The primary aim of the new methodology is to define, in more detail, areas of species richness for conservation planning.

**Methodology:**

Using Maxent, SDMs based on a suite of 14 environmental predictors were generated for 185 West Central African Leguminosae species, each categorised according to one of five vegetation types: Afromontane, coastal, non-flooded forest, open formations, or riverine forest. The relative contribution of each environmental variable was compared between different vegetation types using a nonparametric Kruskal-Wallis analysis followed by a *post-hoc* Kruskal-Wallis Paired Comparison contrast. Legume species diversity patterns were explored initially using the typical method of stacking all SDMs. Subsequently, five different ensemble models were generated by partitioning SDMs according to vegetation category. Ecological modelers worked with legume specialists to improve data integrity and integrate expert opinion in the interpretation of individual species models and potential species richness predictions for different vegetation types.

**Results/Conclusions:**

Of the 14 environmental predictors used, five showed no difference in their relative contribution to the different vegetation models. Of the nine discriminating variables, the majority were related to temperature variation. The set of variables that played a major role in the Afromontane species diversity model differed significantly from the sets of variables of greatest relative important in other vegetation categories. The traditional approach of stacking all SDMs indicated overall centers of diversity in the region but the maps indicating potential species richness by vegetation type offered more detailed information on which conservation efforts can be focused.

## Introduction

The spatial distribution of an organism forms a fundamental basis for studies of biogeography, evolution, patterns of biodiversity, effects of climate change, and invasive species as well as conservation planning, the designation of protected areas, ecological modeling, and statistical or correlative modeling [1]–[4]. Nevertheless, species distributions are often poorly known, especially in tropical areas [5]–[6]. Numerous factors may influence species distribution. In this study, we used statistical and/or correlative species distribution models (SDMs) based on a suite of environmental independent variables to predict the suitability of a given species to an area or areas for which distributional data are either scarce or do not exist [7].

SDMs can be generated using a number of different techniques, each of which is designed to establish a relationship between different environmental variables and available distribution data for a given organism. Commonly, this distribution information is limited to that provided by natural history collections, such as herbarium data. Although these collections record locations where a species has been observed, they rarely provide information on confirmed absences. Other drawbacks associated with herbarium data are sampling bias [8]–[10] and the unknown reliability of georeferences and species identification [11]–[12]. However, the use of well-studied, carefully selected “indicator” taxonomic groups, can allow the identification of conservation targets [13], facilitate data integrity and minimize the impact of SDMs drawbacks [14]–[15]. Unfortunately, there is no consensus as to which indicator groups should be used [16]–[18], and some studies using different indicators offer conflicting biodiversity patterns. For example Howard et al [14] found little spatial congruence in the species richness of woody plants, large moths, butterflies, birds and small mammals across 50 Ugandan forests, but other studies, such that by Urbina-Cardona and Flores-Villela [15] found overlap among the main selected areas in the conservation-area network prioritized to preserve amphibian and reptile species in Mexico.

In this study, we selected Leguminosae (the legumes) as an indicator of angiosperm diversity, [13], [19] (see Materials and Methods). As the third most species-rich angiosperm family, the legumes have been demonstrated as one of the families whose species diversity is best correlated with overall patterns of angiosperm species diversity [19]. First we established an interdisciplinary working group comprised of ecological modelers and specialists in the taxonomy and biology of Leguminosae (M.d.l.E., B.M. and J.J.W. are taxonomists who focus on legumes; R.G.M. and J.M. are SDM experts). Cooperation between taxonomists and ecologists is now considered by many researchers to be an essential element of ecological modeling [20]–[21]. Expert botanical and zoological knowledge can be applied to obtain reliable data, verify the accuracy of identifications, and confirm collection localities, and such knowledge is critical for the biological interpretation and validation of final results [18], [22]–[23]. Moreover, this combined approach has been used to counteract the tendency of many SDMs towards over prediction [3], [24]. However, we acknowledge that some authors [25]–[26] interpret such over prediction as indicating the “fundamental niche” of the species. Indeed, in some cases, apparent over predictions have lead to the detection of new populations of rare taxa or even the discovery of new species or populations of rare species [27]–[29].

Nevertheless, expert knowledge can moderate the limitations of SDMs which arise from their being derived exclusively from climatic or environmental data. These data do not take into account factors such as biotic relationships with other species, limitations of dispersal capacity, historical factors, or the use of complex environmental variables. Although such factors are biologically sound and robustly informative of the organism being modeled [7], [30]–[33], they are difficult to generate. When examining an SDM, taxonomists can consider all these factors and hopefully, in doing so, can ensure that the results more closely reflect the realized niche of the species [34].

When analyzing diversity patterns it is necessary to generate models at community level. Ferrier and Guisan [35] described three strategies: 1) “assemble first, predict later”, in which collections data are classified first, and arranged or aggregated later, e.g.: [36]; 2) “predict first, assemble later”, in which species are modeled singly and the species maps are ordinated or aggregated after, e.g.: [37]; and 3) “assemble and predict together”, in which species are modeled and aggregated at the same time, e.g.: [38]. Many published assessments of the global threat to biodiversity have been based on a species-ensemble approach [39]–[41]. Fewer studies have evaluated the utility of the second strategy [21], [42]–[45].

We also explored another potentially informative approach when developing and interpreting SDMs. We considered the information provided within specimen labels and taxonomic treatments on the vegetation types or formations in which those species has been found (see Material and Methods). Additionally, we sought to elucidate whether species from different vegetation types required different combinations of input variables to be correctly modeled. If that were so, it would be more appropriate to stack the models according to different vegetation type than add all of the available species to the same community-level model. Therefore, we grouped the different species according to their vegetation types before developing the models of potential species richness. To our knowledge, this is the first reported use of this strategy to group species and obtain comparative models of potential species richness according to vegetation type.

The objective of our study was to answer the following three questions: According to the SDMs, what are the diversity patterns of legumes in West Central Africa? Are those diversity patterns in agreement with the current expert opinion? Are the relative contributions of variables to SDMs dependant on the biology (characterized here as their vegetation type preferences) of the species modeled? To answer these questions, the study was conducted in several stages: 1) the creation of the most authoritative and comprehensive legume database for West Central Africa; 2) the use of this database to develop SDMs for individual species and the subsequent stacking of individual models to generate models of potential species richness; 3) the investigation of the relative influence of the independent variables in the generation of accurate models of species of different vegetation types; 4) a comparison of the reliability of diversity patterns obtained for each vegetation type; and 5) the assessment of the generated SDMs by taxonomic experts from both biological and conservation perspectives.

## Materials and Methods

### Study area: choice of geographical delimitation and taxonomic focus

**Figure 1:**
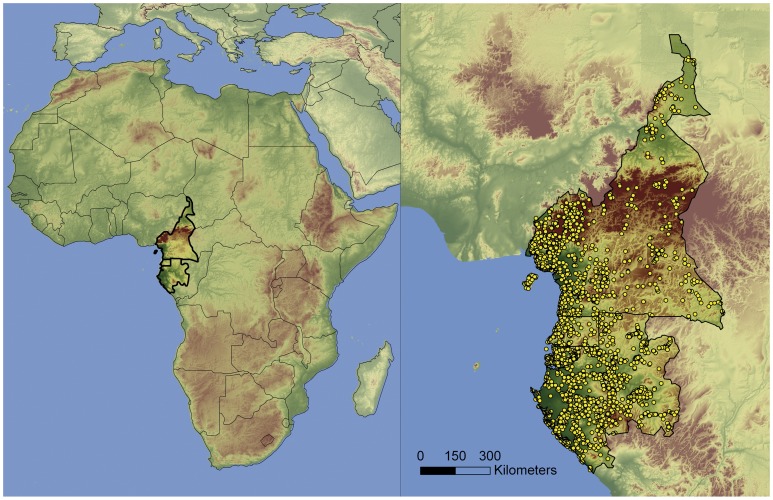
Study area and occurrences of the 185 species used to generate the species distribution models.

West Central Africa represents the area of greatest biodiversity within tropical Africa [46]–[47]. Within the region, the botany of Cameroon, Gabon, and Equatorial Guinea is relatively well-explored. Their floras have been and continue to be a research focus for several legume taxonomic specialists, e.g.: [48]–[57]. In addition to the mainland territories, we also included Bioko Island, the largest island of the Gulf of Guinea (2,017 km^2^). Although Bioko is administratively part of Equatorial Guinea, it lies only 32 km west of the Cameroon coast. This island is under significant continental influence as evidenced by the flora, which is quite similar to that of the mainland [58]. The other three islands within the Gulf of Guinea belong to the same volcanic arc as Mount Cameroon and Bioko, but are much smaller in size. They consist of Príncipe (114 km^2^), São Tomé (857 km^2^), and Annobón (17 km^2^) and are not included in the present study (Figure 1).

### Study group

Leguminosae is the third largest family of flowering plants comprising approximately 19,300 species recognized in three subfamilies, Caesalpinioideae, Mimosoideae, and Papilionoideae. The legumes occur in a great variety of vegetation formations from rainforests and mangrove swamps to deserts and temperate or alpine zones [59]. In economic terms they are arguably the most important family of plants [59]–[62]. Furthermore, many species have the capacity to colonize otherwise barren lands through symbiotic fixation of atmospheric nitrogen in their root nodules [63]. In terms of species richness, Leguminosae is the most important angiosperm family of tropical African forests [64]. Of the three subfamilies, Caesalpinioideae is the smallest group [59], [65] comprised of c. 2,250 species. Caesalpinioid legumes have a primarily tropical distribution and typically bear large, showy flowers. Many of the tree species in Africa belong to this subfamily, where they are the most dominant taxonomic group of flowering plants in lowland evergreen rainforest [57]. The Mimosoideae subfamily has a slightly larger number of species (c. 3,270 species), which are also most commonly found in the tropics. Mimosoid legumes are not well represented in the rainforest and generally prefer drier vegetation formations. Typically, they have small flowers aggregated into heads or spikes. The mimosoid legume genera include widely recognized species-rich genera such as *Mimosa* and *Acacia*. The cosmopolitan Papilionoideae is by far the largest legume subfamily with c. 13,800 species. Papilionoideae is a generalist taxon with respect to vegetation formations, and papilionoid legumes often bear characteristic “pea” flowers [59].

### Herbarium specimen data

We assembled a database containing 16,780 legume records from Cameroon, Equatorial Guinea, and Gabon by merging the databases of specimens kept in three herbaria: WAG (Wageningen University, Wageningen), K (Royal Botanic Gardens, Kew), and MA (Real Jardín Botánico, Madrid). After these data were merged, legume specialists verified the accuracy of the taxonomic identifications and geographical localities of the specimens. Any records that were in doubt were excluded. Furthermore, to avoid the influence of species misidentification, we excluded from the dataset genera that are currently under taxonomic study [66]–[69]. These are taxa where species and/or generic limits are not yet resolved, e.g., the genera *Hymenostegia* and *Gilbertiodendron* are presently under revision by Mackinder & Wieringa and Estrella & Devesa, respectively. We also excluded species that were introduced and likely naturalized in our study area. Included collections were placed on a 0.0083° (c.1 km) geographic grid. When multiple collections of the same species occurred within the same pixel, a single presence was recorded. SDMs with few occurrences are generally less accurate [70]–[72]; thus, only species with at least 15 unique presences were modeled to avoid generating low-performance models. Total specimens and different localities for each species analyzed are indicated within Table S1. We chose a cutoff of 15 presences based on recommendations from other studies [73]–[74]. The final edited database included 7,445 records of 185 species: 87 species from 41 genera of the Caesalpinioideae, 24 species from 15 genera of the Mimosoideae, and 74 species from 39 genera of the Papilionoideae (Table S1, Figure 1).

Each of the 185 species was assigned to a vegetation type using data that were extracted primarily from taxonomic studies (references under study area; Table S1). Those data were supplemented by field observations recorded on herbarium specimen labels after they had been reviewed by taxonomic experts for anomalies. Each species was assigned to one of five categories; Afromontane (AF), coastal (CO), non-flooded forest (NF), open formations (OF) or riverine (RF) vegetation type. Species that had been documented as present in more than one vegetation type were assigned to the most frequently reported category (Table S1).

### Environmental predictors

To obtain the bioclimatic variables, we employed the widely used World Clim 1.4 dataset [75] (http://www.worldclim.org) at a 1×1 km spatial resolution. Because one of the purposes of this study was to explore the relative contribution of the bioclimatic variables, we performed a Pearson's pairwise correlation analysis in SPSS (www.spss.com) and removed one of the variables in each pair that had a pairwise correlation value higher than 0.8; the removed variable of each pair was thus considered to be the less biologically important of the two, considering the legumes as a whole. However, we acknowledge that in some cases both were similarly important, and our decision to drop one was arbitrary in biological terms, but needed to avoid multicollinearity. The final variable set included bio_02, bio_03, bio_06, bio_08, bio_09, bio_16, bio_17, bio_18 and bio_19 (Table 1).

**Table 1:** Independent variable codes and explanations. Codes prefixed by “bio_” were derived from WORLDCLIM 1.4; sources of other variables are described in the text.

Variable	Code	Description of variable
1	bio_02	Mean Diurnal Range [Mean of monthly (max temp – min temp)]
2	bio_03	Isothermality (P2/P7) (* 100)
3	bio_06	Min Temperature of Coldest Month
4	bio_08	Mean Temperature of Wettest Quarter
5	bio_09	Mean Temperature of Driest Quarter
6	bio_16	Precipitation of Wettest Quarter
7	bio_17	Precipitation of Driest Quarter
8	bio_18	Precipitation of Warmest Quarter
9	bio_19	Precipitation of Coldest Quarter
10	Distance	Distance to the sea shore
11	Eastness	Orientation E-W
12	Geology (soils)	Categorical variable with the soil information
13	Northness	Orientation N-S
14	Compound Topographic Index	potential water accumulation

In addition to bioclimatic variables, we generated a variable indicating the distance to the sea and characterized the topography, slope and aspect via two compound variables derived from a 250 m resolution DEM (http://srtm.csi.cgiar.org/):

“northness” = cosine (aspect in radians) = cosine (aspect * 3.14159/180)

“eastness” = sine (aspect in radians) = sine (aspect * 3.14159/180)

Flat terrain was reclassified as 0.

We also derived a Compound Topographic Index from the 250 m DEM using an ArcInfo Workstation (cti.aml script, available at http://arcscripts.esri.com). The Compound Topographic Index is a function of both the slope and the upstream catchment area and can be considered a measure of the potential water accumulation, which is useful for modeling species related to watercourses.

Finally, we used the Map of Geologic Provinces of Africa 2.0 (U.S. Geological Survey) to obtain the geologic data.

### Ecological modeling

Species distribution models were generated in Maxent 3.3.3e with the default settings (“Auto features”, convergence threshold = 10^−5^, maximum number of iterations = 500, maximum number of background points = 10,000, regularization multiplier = 1). Due to the low sample size of most of the species, as is commonly the case in tropical species studies [5], [24], data resampling is not the best strategy for those data [37]. We conducted a verification of the models using 100% of the data as the training data set [34]; AUC values calculated from a limited number of presences can lead to a prediction of model accuracy that is artificially high, compared to a prediction calculated from a more complete knowledge of the potential distribution [76].

The “maximum training sensitivity plus specificity” rule was used to convert the resulting continuous models to binary models.

Individual binary SDMs were combined to generate six models of potential species richness, one for all of the species and five for the considered vegetation types: (1) Total species, (2) non-flooded forest, (3) open formations, (4) riverine, (5) coastal, and (6) Afromontane. Those individual species models, as well as the stacked vegetation type models were analyzed for consistency with published patterns of legume richness in West Central Africa, e.g.: [47], [77]–[82].

### Relative contributions of the environmental variables

Maxent provides an estimate of the relative contribution of each environmental variable to the generated SDM model [83] (Table S1). We used those relative contributions as variables to determine how, if at all, contributions of the environmental variables differed across vegetation types. Because the assumptions of normality and homoscedasticity were not met, we performed a nonparametric Kruskal-Wallis test using the vegetation type as a grouping variable (AF, CO, OF, NF, and RF) followed by a post-hoc Kruskal-Wallis Paired Comparisons analysis [84] (http://www.brightstat.com). Finally, we performed a non-metric multidimensional scaling (NMDS) analysis of the contributions of the environmental variables to explore whether taxa that appears at the same vegetation type would group according to the variable contribution, for this analysis R and the Vegan package were used (http://cran.r-project.org/web/packages/vegan/index.html; http://cc.oulu.fi/~jarioksa/opetus/metodi/vegantutor.pdf).

## Results

### Maxent models

The 185 species models generated with Maxent had accuracy values, measured as the Area Under the ROC Curve (AUC), from 0.84 to 0.99 for the training data set. Values in this range are considered indicative of good accuracy [85].

### Vegetation types analyses

According to the Kruskal-Wallis test, the following environmental variables exhibited different contributions to the models across vegetation types (*P*<0.01): the mean diurnal range (bio_02), isothermality (bio_03), mean temperature of wettest quarter (bio_08), mean temperature of driest quarter (bio_09), precipitation of wettest quarter (bio_16), precipitation of driest quarter (bio_17), precipitation of warmest quarter (bio_18), precipitation of coldest quarter (bio_19), distance, eastness, geology, northness and Compound Topographic Index (CTI) (Table 2).

**Table 2:** The results of the Kruskal-Wallis test (***, *p*<0.001).

Variable	n tied Ranks	Chi-Square	df	p (2-tailed)
bio_02	61	25.76633	4	0,0004***
bio_03	38	31.84419	4	0.0000***
bio_06	41	6.22594	4	0.1829
bio_08	54	29.1088	4	0,0001***
bio_09	53	35.98039	4	0.0000***
bio_16	81	15.56136	4	0.0037***
bio_17	2	50.05549	4	0.0000***
bio_18	12	42.79366	4	0.0000***
bio_19	6	23.10991	4	0.0001***
Distance	2	84.01005	4	0.0000***
eastness	5	13.86386	4	0.0077***
geology	0	61.58766	4	0.0000***
northness	3	17.67899	4	0.0014***
Compound Topographic Index (CTI)	6	30.85877	4	0.0000***

According to the post-hoc Kruskal-Wallis Paired Comparisons test (Table 3, Table S2); the set of variables that contributed most strongly to the Afromontane species models was significantly different from that of the other vegetation types. Contributions from precipitation of driest quarter (bio_17), eastness, northness and Compound Topographic Index (CTI) were considerably more important to Afromontane species models than to those of open formation taxa. A more complex set of variables separated Afromontane species from CO, NF and RF taxa, including the mean temperature of the wettest quarter (bio_08), distance and geology (Table 3). In the case of coastal species, distinguishing variables included bio_17, distance and CTI. Species classified as OF were separated from NF and RF species based on geologic factors. Finally, NF species differed from RF taxa based on the precipitation of the warmest quarter (bio_18) (Table 3).

**Table 3:** Highly significant comparisons (*p*<0.001) of the Kruskal-Wallis Paired Comparisons [84].

Variable	I	J	5% Critical Difference	(I-J) Differences	Variable	I	J	5% Critical Difference	(I-J) Differences
bio_02	AF	RF	3.251.608	−7.062.805	bio_19	AF	CO	3.465.914	7.248.148
	OF	RF	2.513.928	−5.231.805			RF	3.278.788	5.685.772
	RF	AF	3.251.608	7.062.805		CO	AF	3.465.914	−7.248.148
		OF	2.513.928	5.231.805			NF	2.223.428	−3.926.065
bio_03	AF	NF	3.007.433	−6.337.917		NF	CO	2.223.428	3.926.065
	CO	NF	2.162.234	−4.930.972		RF	AF	3.278.788	−5.685.772
	OF	NF	222.596	−407.075	distance	AF	CO	2.732.319	−14.875.463
	NF	AF	3.007.433	6.337.917			NF	2.437.978	−7.274.792
		CO	2.162.234	4.930.972			RF	25.848	−8.793.801
		OF	222.596	407.075		CO	AF	2.732.319	14.875.463
bio_08	AF	CO	3.400.686	943.287			OF	2.185.855	10.336.296
		NF	3.034.345	7.159.583			NF	1.752.817	7.600.671
		RF	3.217.082	7.399.492			RF	1.951.876	6.081.662
	CO	AF	3.400.686	−943.287		OF	CO	2.185.855	−10.336.296
	NF	AF	3.034.345	−7.159.583			RF	1.998.396	−4.254.634
	RF	AF	3.217.082	−7.399.492		NF	AF	2.437.978	7.274.792
bio_09	AF	CO	3.324.396	8.138.889			CO	1.752.817	−7.600.671
		NF	2.966.274	7.499.167		RF	AF	25.848	8.793.801
	CO	AF	3.324.396	−8.138.889			CO	1.951.876	−6.081.662
		OF	2.659.517	−5.102.222			OF	1.998.396	4.254.634
	OF	CO	2.659.517	5.102.222	eastness	AF	OF	3.607.712	−6.220.333
		NF	2.195.496	44.625		OF	AF	3.607.712	6.220.333
	NF	AF	2.966.274	−7.499.167	geology	AF	CO	3.023.192	11.892.593
		OF	2.195.496	−44.625			NF	2.697.517	91.9
bio_17	AF	OF	3.201.076	−6.073.667			RF	2.859.969	7.656.098
		NF	2.821.721	−5.699.167		CO	AF	3.023.192	−11.892.593
	CO	OF	2.529.913	−7.750.519			OF	2.418.554	−8.240.593
		NF	2.028.714	−7.376.019			RF	2.159.666	−4.236.495
		RF	2.259.105	−6.645.348		OF	CO	2.418.554	8.240.593
	OF	AF	3.201.076	6.073.667			NF	1.996.575	55.38
		CO	2.529.913	7.750.519			RF	2.211.139	4.004.098
	NF	AF	2.821.721	5.699.167		NF	AF	2.697.517	−91.9
		CO	2.028.714	7.376.019			OF	1.996.575	−55.38
	RF	CO	2.259.105	6.645.348		RF	AF	2.859.969	−7.656.098
bio_18	AF	NF	2.897.202	−8.482.292			CO	2.159.666	4.236.495
	CO	NF	2.082.982	−4.669.792			OF	2.211.139	−4.004.098
	OF	NF	2.144.372	−3.821.125	northness	AF	OF	3.567.033	−6.170.667
	NF	AF	2.897.202	8.482.292		CO	OF	281.914	−4.807.704
		CO	2.082.982	4.669.792		OF	AF	3.567.033	6.170.667
		OF	2.144.372	3.821.125			CO	281.914	4.807.704
		RF	179.753	4.309.223	CTI	AF	OF	3.422.785	−69.68
	RF	NF	179.753	−4.309.223			RF	3.198.857	−6.632.927
		NF	2.169.224	−3.716.597		CO	OF	2.705.137	−5.790.222
		RF	2.415.572	−5.455.149			NF	2.169.224	−3.716.597
	OF	AF	3.422.785	69.68			RF	2.415.572	−5.455.149
		CO	2.705.137	5.790.222		OF	AF	3.422.785	69.68
	NF	CO	2.169.224	3.716.597			CO	2.705.137	5.790.222
	RF	AF	3.198.857	6.632.927		NF	CO	2.169.224	3.716.597
		CO	2.415.572	5.455.149		RF	AF	3.198.857	6.632.927
							CO	2.415.572	5.455.149

AF, Afromontane species; CO, Coastal species; OF, open formations; NF, non-flooded forest; RF, riverine or water-associated species. I and J, comparison of formation pairs (for all comparisons see Table S2).

**Figure 2:**
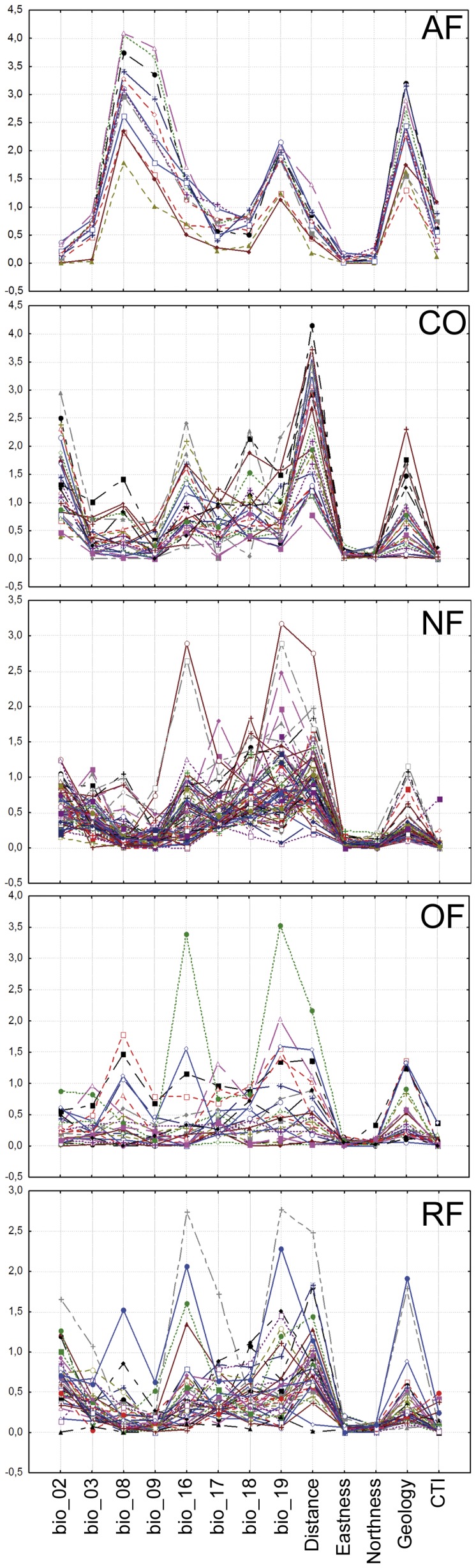
Maxent jackknife test of variable importance. Each curve represents the regularized training gain of each variable used in isolation for each species. AF, Afromontane species; CO, coastal species; NF, non-flooded forest species; OF, open formations species; RF, riverine species.

According to the jackknife test of variable importance (Figure 2; Table S1), the most important variables in the AF species model were bio_08, bio_09, and geology. For the CO taxa, distance to seashore, geology, bio_02, and bio_16 were most important. For the NF taxa, distance to seashore, bio_19, bio_18, bio_02, and bio_16 made the greatest contributions. For the RF taxa, distance to seashore, bio_02, bio_19, and bio_18 played the greatest roles. Finally, for the OF species, distance to seashore, bio_19, geology, and, bio_16 were most important.

**Figure 3:**
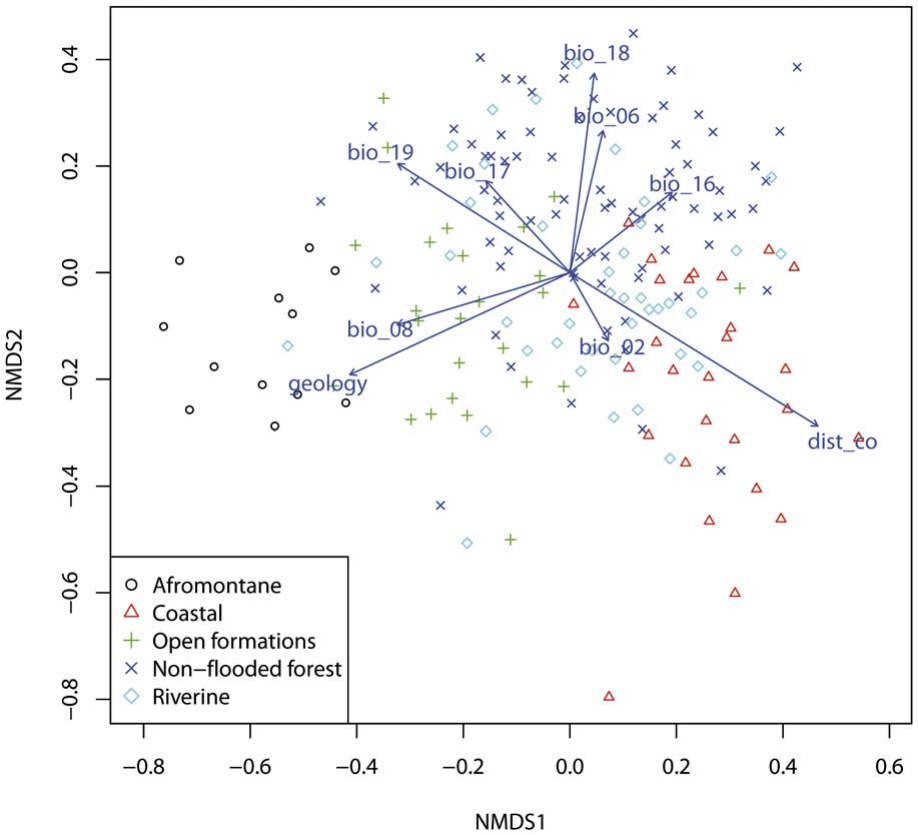
Non-metric multidimensional scaling (NMDS) of the variable contributions for each species distribution model (SDM). Each species is represented within the relevant vegetation type classification; arrows indicate the direction of the maximum variable contribution for the SDMs.

The results of the NMDS analysis show that geology and bio_08 were the most important variables contributing to the SDMs of AF taxa, distance to seashore was the most important variable for the coastal taxa and bio_18, bio_06 and bio_16 were the most important variables for NF species (Figure 3).

### Legume diversity patterns

**Figure 4:**
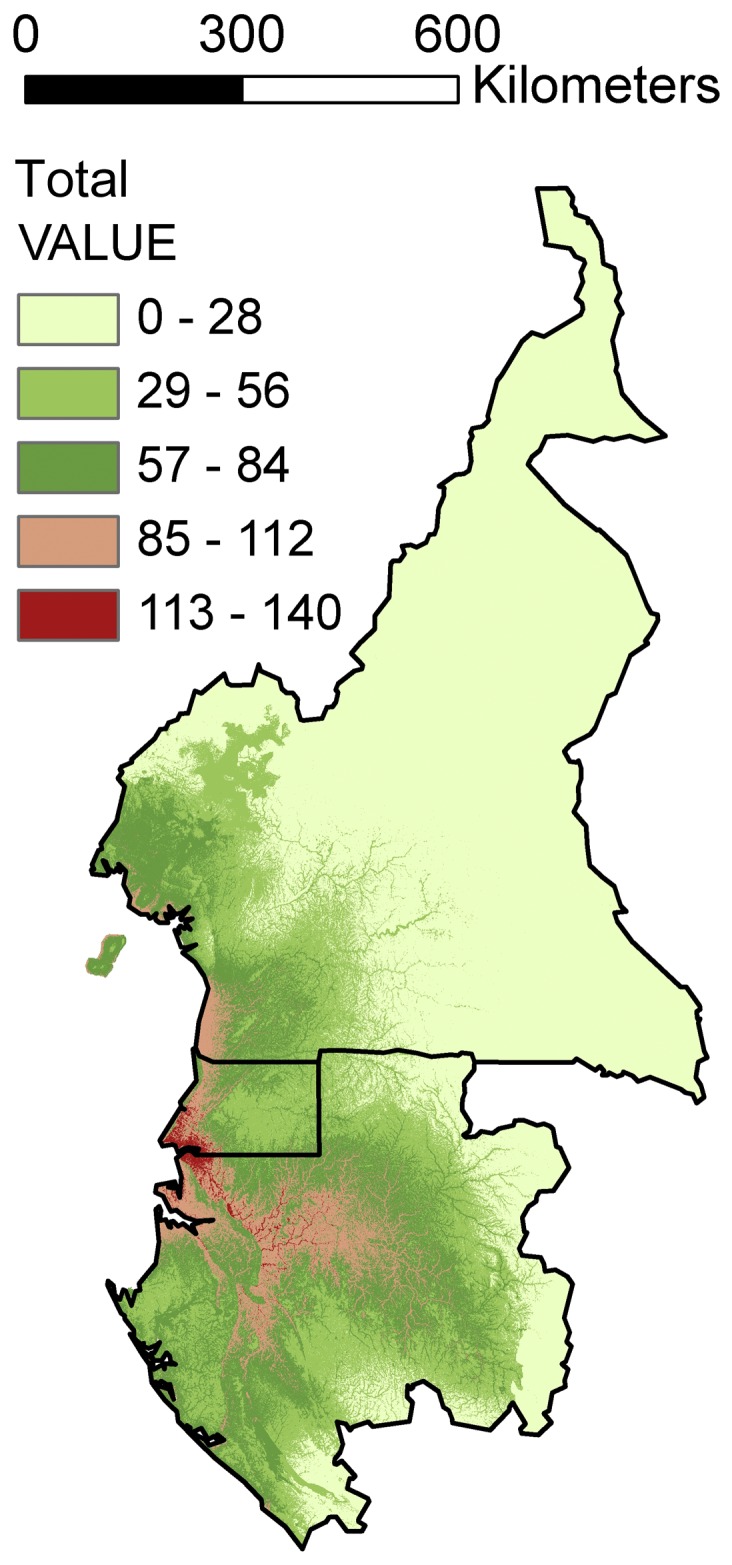
Potential species richness map for the stacked model of all studied species.

**Figure 5:**
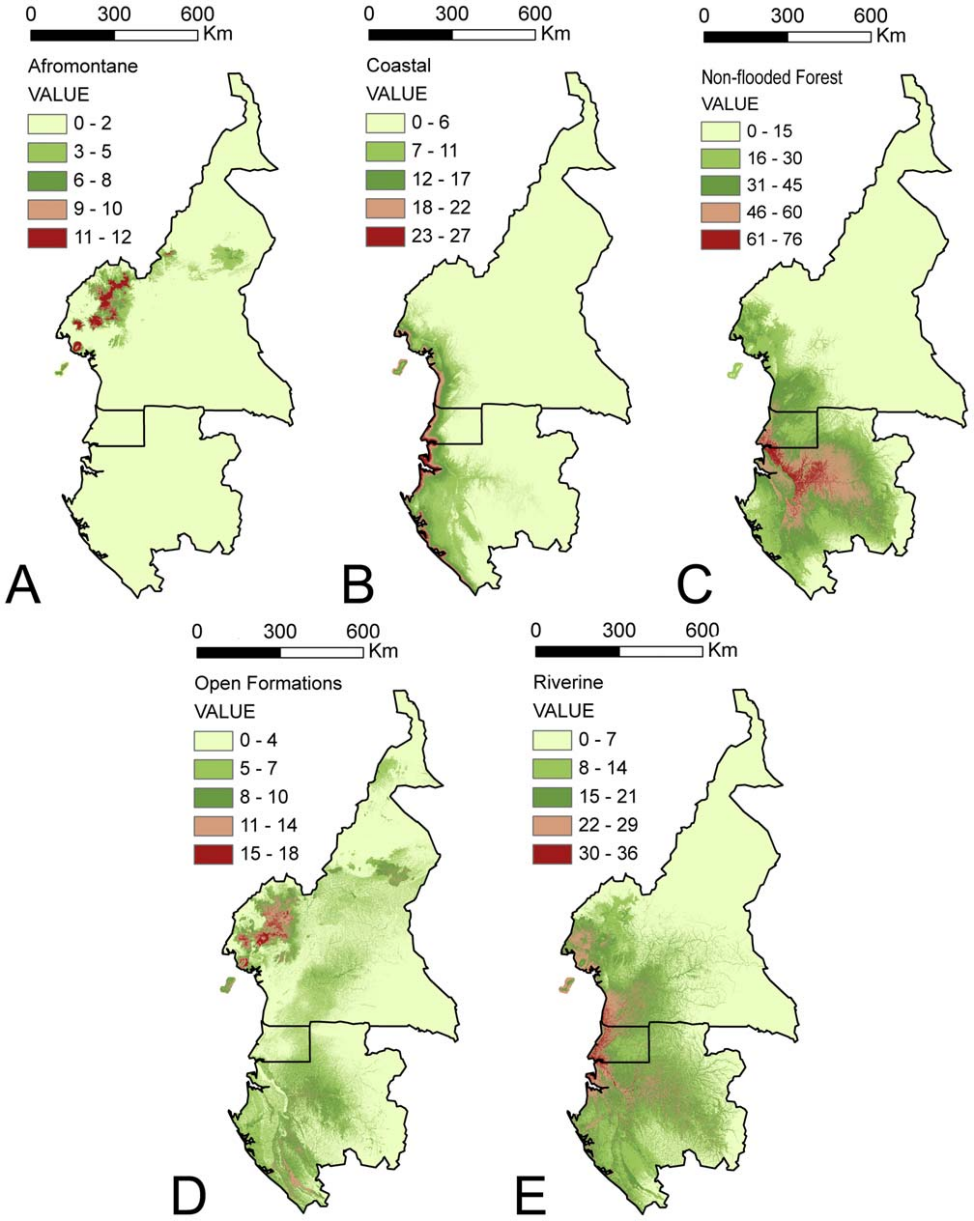
Potential species richness maps according the different vegetation types. (a) Afromontane species, (b) coastal species, (c) non-flooded forest species, (d) open formations species, and (e) riverine species.

Individual models within each reported vegetation types were stacked to generate vegetation richness models. One hundred and eighty-five species were included in the general model, and the Afromontane, coastal, non-flooded forest, open formations, and riverine models each contained 12, 27, 80, 25 and 41 species, respectively (Table S1; Figures 4 and 5A–E).

## Discussion

### Vegetation type analysis

The vegetation type analysis allowed us to discern which of the environmental factors used in this study were the most appropriate variables for modeling suitability of vegetation types for Leguminosae in West Central Africa. Although the variables used here are only a portion of all parameters that could be used, they are among the most commonly employed variables in ecological modeling [86]. Many of them represent limiting factors for legume distribution ranges in tropical Africa. The generation of other variables can be difficult or even impossible for tropical areas. Satellite-derived parameters, although widely used, may not represent biologically important characteristics. Moreover, they can be difficult to correlate with biological characteristics or have unsuitable spatial resolution (e.g., LAI and QSCAT backscatter data).

The majority of important bioclimatic variables for discriminating among vegetation types were related to temperature variations (Table 2). In tropical Africa, water availability has been suggested as the most important factor explaining the distribution of individual plant species [87]–[88], the water deficit is a function of rainfall and evaporation (which depends on temperature, humidity and wind). Precipitation variables are relatively important, but mainly because of seasonality which is also related to the water deficit (Table 2).

Afromontane species were readily separated (Table 3, Figure 3) from species of all other vegetation types based on the mean temperature of the wettest quarter (bio_08) and the geology. Afromontane taxa grow at altitudes exceeding 2500 m on volcanic mountains with temperatures and precipitation similar to temperate regions; thus, it is reasonable that those variables were the most important parameters in the generated models (Figure 2). For example, species of mostly temperate genera, e.g., *Trifolium* and *Adenocarpus*, are found in Afromontane vegetation which has a widely disjunct distribution pattern on the high mountains of tropical Africa.

For coastal taxa, distance to sea shore was the most important variable (Table 3, Figures 2 and 3). It is logical that the SDMs of such species, which are adapted to high humidity and a coastal influence, were highly responsive to this variable.

“Non-flooded forest” species were separated (Table 3) from AF and RF taxa by variables defining periods of water shortages, i.e., precipitation of the driest quarter (bio_17), precipitation of the warmest quarter (bio_18), as well as distance to seashore. The separation of “non-flooded forest” taxa from OF taxa was also distinct (Figure 3) and was mainly based on the mean temperature of the driest quarter (bio_09), precipitation of the warmest quarter (bio_18), and the geology. Figure 3 illustrates that there was not a clear-cut limit between open formations, non-flooded forests, and riverine vegetation types but that rather a gradient of change was observed. The non-flooded forests vegetation type includes several different vegetation sub-types, such as primary lowland dry forest, periodically inundated forest, or primary forest on white sands. Although these vegetation types are not clearly defined or fully independent from one another, the inclusion of these groups may explain the difficulty in identifying distinct groups in Figure 3. This effect is likely the consequence of the near impossibility of classifying many of the species typical of the NF vegetation into fully discrete categories.

Within the open formations (OF) category, we included species from savannah and lowland grasslands, which appear in the mountains at lower altitudes than the Afromontane species and also occur on volcanic soils. The inclusion of these vegetation types explains the clear separation of OF from NF and RF taxa based on geology (Figure 3, Table 3). Variables related to the seasonality of the precipitation, i.e. precipitation of the driest quarter (bio_17) and precipitation of the warmest quarter (bio_18), were also important in OF species models (Figures 2 and 3).

The models for species of riverine forests (RF) indicated that the distance to seashore and the Compound Topographic Index (CTI), a surrogate for water accumulation, played an important role (Figure 2 and 3). The CTI was less important for modeling RF taxa than the precipitation of the coldest quarter (bio_19) and the geology, variables also related to rivers and water sources (Figure 2). However, the CTI remains a crucial variable when generating RF taxa models; for instance the SDM of *Aphanocalyx djumaensis*, a gregarious species of riverine forest, showed that CTI was the most important variable in the result of the jackknife test (Table S1). Global bioclimatic variables were able to determine the general distribution pattern of a species, although the quality of predictions was improved when other variables representing edaphic factors were included.

### Legume diversity patterns in West Central Africa

As a first approach, we stacked all of the SDMs (Figure 4) as has been performed in other studies investigating centers of diversity or conservation priorities, e.g.: [43], [89]–[90]. The previous vegetation types analysis revealed that there are differences in model predictions and that the influence of independent variables is dependent on the vegetation type. This information led us to generate ensemble models by stacking together only those species living in the same vegetation rather than all species.We generated five stacked maps, one for each vegetation category: Afromontane (Figure 5A), coastal (Figure 5B), non-flooded forest (Figure 5C), open formations (Figure 5D) and riverine (Figure 5E).

The potential species richness map for Afromontane species (Figure 5A) clearly illustrates that this vegetation type is restricted to the highest mountains of Bioko Island, Mount Cameroon and the Adamawa Mountains, all of which belong to the same volcanic arc. This was the expected distribution for this vegetation type despite the small number of studied species. Afromontane species share a well-defined set of environmental conditions. As a consequence, this vegetation type can be accurately captured with fewer presence points than for generalist taxa [73], [91]–[93]. We also observed that Afromontane species present a common jackknife curve pattern for all of the species that contrasted with the more variable jackknife curve patterns obtained from species of the other vegetation types.

Figures 5B and 5E display the stacked maps for coastal and riverine species, respectively. Coastal vegetation (Figure 5B) is endangered throughout the world, and our results indicate that the southern coast of Bioko Island and the entire coast of Gabon should be considered in future conservation planning efforts.

Non-flooded forests (Figure 5C) are potentially more suitable for conservation in the low territories of the Rio Campo region in Cameroon, the Muni estuary in Equatorial Guinea, and the Ogooué basin in Gabon. This prediction corresponds to the area which currently has the largest expanse of pristine forests in the continent [46]–[47], [57], and would indicate this area as a priority for conservation programs in tropical Africa. These forests are dominated by members of the subfamily Caesalpinioideae, particularly in the vicinity of the Muni estuary, the Ogooué river basin in Gabon, and around Kribi in Cameroon, an area with a dense Caesalpinioideae forest in good condition. The lowlands of Bioko Island, the most populated and disturbed part of the island and thus the area that has been transformed into secondary vegetation, also appears to have suitable primary vegetation. For example, the Gran Caldera de Luba, located in the southern area of the island and surrounded by an expanse of secondary forests, holds large patches of pristine forest. Secondary vegetation types are also dense in Cameroon north of Mount Cameroon and near the villages of Bafousam and Bamenda, both densely populated areas, and in Gabon near the capital city of Libreville, likely one of the most altered areas of the country. These areas should be targets for future conservation planning strategies, although we acknowledge that anthropic pressure can lead to social conflicts resulting in the failure of such efforts.

A similar pattern to that of the Afromontane taxa was found for the open formation species (Figure 4B), which are more abundant at lower altitudes than Afromontane species. The Open Formation species have an important presence at the coast from Cameroon to Gabon and on Bioko Island where coastal grasslands on sand are a highly endangered vegetation type. The savannah species are important in northern Cameroon near Ngaoundere and in southern Gabon in the Moukalaba-Doudou reserve.

We hypothesize that the biology of the species is a critical consideration when deciding whether models of different taxa should or should not be stacked. Such a decision should also take into account the objectives of a study. If our goal was to preserve the highest areas of legume diversity, we would use the total stack option (Figure 4). This would indicate that the most important areas are those surrounding Mount Cameroon, the Kribi area of Cameroon, the Muni estuary in Equatorial Guinea, and the Ogooué basin in Gabon. Most modeling exercises stack the species distribution models of all available species irrespective of the biological implications; unfortunately, this approach may result in the loss of important information. We advocate the strategy of stacking species of similar vegetation type because it can lead to better-defined areas of species richness on which conservation priorities may be based. Specifically, the most species-rich areas of primary and riverine forest were correctly identified (Figure 5C–E). These forest types are globally threatened by increasing population and should be primary targets for conservation planning in the region. The Afromontane eco-region of Pico Basilé, Mount Cameroon and the Adamawa mountains was also appropriately delimited by models, as were the grasslands located at lower altitudes in the mountains and savannahs (Figure 5D) of northern Cameroon and southern Gabon. Coastal areas (Figure 5B) with extensive mangroves, another globally threatened vegetation type [94], were also well captured.

### Conclusions

The capacity of SDMs to reproduce patterns of species richness has been demonstrated before [37], but should be used with caution. In particular, when modeling species richness, an indiscriminate use of all species in the database, e.g., the use of secondary vegetation type or introduced species for the assessment of conservation priorities, will likely lead to errors. Care should be taken in selecting species and independent variables appropriate to the purposes of the study. Additionally, the biology of the species should be considered. To increase the accuracy of the obtained SDMs, future works should strive to incorporate species dispersal capacity, interactions between species, or geographical barriers into model development. We obtained better-defined potential species-rich areas when we stacked species of similar vegetation type than those obtained by stacking all species in the study group irrespective of vegetation type. Future studies comparing species with similar jackknife curves would be of value. The accurate modeling of Afromontane species in this study supports the findings of previous works that suggest that SDMs better reflect the distribution patterns of species with restricted distributions [73]–[74]. The common jackknife pattern found in AF species (Figure 2) could be indicative that a selected group of species are characterized by a well-defined set of environmental parameters. Jacknife patterns were more variable in the other vegetation types.

We conclude that it is essential to select an appropriate group of independent variables to correctly model species distributions; thus, any knowledge of the biology of the modeled species is highly desirable when developing SDMs and can improve the accuracy and reliability of the final outcome. We have demonstrated that the role of a bioclimatic variable in a SDM differs between vegetation types. Our experience indicates that knowing the biology of a species can assist in selecting variables with good predictive power.

Floristic knowledge in Africa is far from complete, and extensive gaps in the available distribution data represent a serious impediment to completing our knowledge of broad-scale patterns of plant diversity [95]. This hurdle could be overcome in part by combining different datasets to develop SDMs. We recommend that the resultant models be carefully verified by experts who can evaluate the results based on their understanding of the biology of the species being modeled.

Finally, studies similar to the work presented here could be used to guide taxonomists to plan more cost-effective field expeditions, which require considerable effort in terms of both time and money, recent expeditions has been planned using collection density maps [47] and phenology data from databases. SDMs are without doubt a useful tool for maximizing research outcomes within the constraints of all too frequently scarce resources.

## Supporting Information

Table S1 Maxent results for the 185 Leguminosae species studied.
(XLS)

Table S2 The results of the Kruskal-Wallis Paired Comparisons [84]. AF, Afromontane species; CO, Coastal species; OF, open formations; NF, Non-flooded forest; RF, riverine or water-associated species. I and J, comparison of formation pairs (*,*p*<0.05; **,*p*<0.01; ***,*p*<0.001).
(DOC)
