# Supplementary material for: Legume Diversity Patterns in West Central Africa: Influence of Species Biology on Distribution Models
Source: PLoS One. 2012 Jul 23;7(7):e41526. doi: 10.1371/journal.pone.0041526 (PMC3402427; doi:10.1371/journal.pone.0041526)
Supplement: Table S2 — The results of the Kruskal-Wallis Paired Comparisons [84]. AF, Afromontane species; CO, Coastal species; OF, open formations; NF, Non-flooded forest; RF, riverine or water-associated species. I and J, comparison of formation pairs (*,p<0.05; **,p<0.01; ***,p<0.001). (DOC) [file pone.0041526.s002.doc]

**Table S2.** The results of the Kruskal-Wallis Paired Comparisons after [84]. AF, Afromontane species; CO, Coastal species; OF, open formations; NF, Non-flooded forest; RF, riverine or water-associated species. I and J, comparison of formation pairs (*,*p*<0.05; **,*p*<0.01; ***,*p*<0.001).

| **Variable** | **I** | **J** | **5 % Critical Difference** | **(I-J) Differences** | **p** |
| --- | --- | --- | --- | --- | --- |
| bio_02 | AF | CO | 3.437.183 | -5.167.593 | ** |
|  |  | OF | 3.479.229 | -18.31 |  |
|  |  | NF | 3.066.911 | -39.5 | * |
|  |  | RF | 3.251.608 | -7.062.805 | *** |
|  | CO | AF | 3.437.183 | 5.167.593 | ** |
|  |  | OF | 2.749.746 | 3.336.593 | * |
|  |  | NF | 2.204.996 | 1.217.593 |  |
|  |  | RF | 2.455.407 | -1.895.212 |  |
|  | OF | AF | 3.479.229 | 18.31 |  |
|  |  | CO | 2.749.746 | -3.336.593 | * |
|  |  | NF | 2.269.982 | -21.19 |  |
|  |  | RF | 2.513.928 | -5.231.805 | *** |
|  | NF | AF | 3.066.911 | 39.5 | * |
|  |  | CO | 2.204.996 | -1.217.593 |  |
|  |  | OF | 2.269.982 | 21.19 |  |
|  |  | RF | 1.902.824 | -3.112.805 | ** |
|  | RF | AF | 3.251.608 | 7.062.805 | *** |
|  |  | CO | 2.455.407 | 1.895.212 |  |
|  |  | OF | 2.513.928 | 5.231.805 | *** |
|  |  | NF | 1.902.824 | 3.112.805 | ** |
| bio_03 | AF | CO | 3.370.524 | -1.406.944 |  |
|  |  | OF | 3.411.755 | -2.267.167 |  |
|  |  | NF | 3.007.433 | -6.337.917 | *** |
|  |  | RF | 3.188.548 | -5.084.045 | ** |
|  | CO | AF | 3.370.524 | 1.406.944 |  |
|  |  | OF | 2.696.419 | -860.222 |  |
|  |  | NF | 2.162.234 | -4.930.972 | *** |
|  |  | RF | 2.407.788 | -36.771 | ** |
|  | OF | AF | 3.411.755 | 2.267.167 |  |
|  |  | CO | 2.696.419 | 860.222 |  |
|  |  | NF | 222.596 | -407.075 | *** |
|  |  | RF | 2.465.174 | -2.816.878 | * |
|  | NF | AF | 3.007.433 | 6.337.917 | *** |
|  |  | CO | 2.162.234 | 4.930.972 | *** |
|  |  | OF | 222.596 | 407.075 | *** |
|  |  | RF | 1.865.922 | 1.253.872 |  |
|  | RF | AF | 3.188.548 | 5.084.045 | ** |
|  |  | CO | 2.407.788 | 36.771 | ** |
|  |  | OF | 2.465.174 | 2.816.878 | * |
|  |  | NF | 1.865.922 | -1.253.872 |  |
| bio_08 | AF | CO | 3.400.686 | 943.287 | *** |
|  |  | OF | 3.442.286 | 5.425.833 | ** |
|  |  | NF | 3.034.345 | 7.159.583 | *** |
|  |  | RF | 3.217.082 | 7.399.492 | *** |
|  | CO | AF | 3.400.686 | -943.287 | *** |
|  |  | OF | 2.720.549 | -4.007.037 | ** |
|  |  | NF | 2.181.583 | -2.273.287 | * |
|  |  | RF | 2.429.335 | -2.033.379 |  |
|  | OF | AF | 3.442.286 | -5.425.833 | ** |
|  |  | CO | 2.720.549 | 4.007.037 | ** |
|  |  | NF | 2.245.879 | 173.375 |  |
|  |  | RF | 2.487.235 | 1.973.659 |  |
|  | NF | AF | 3.034.345 | -7.159.583 | *** |
|  |  | CO | 2.181.583 | 2.273.287 | * |
|  |  | OF | 2.245.879 | -173.375 |  |
|  |  | RF | 1.882.619 | 239.909 |  |
|  | RF | AF | 3.217.082 | -7.399.492 | *** |
|  |  | CO | 2.429.335 | 2.033.379 |  |
|  |  | OF | 2.487.235 | -1.973.659 |  |
|  |  | NF | 1.882.619 | -239.909 |  |
| bio_09 | AF | CO | 3.324.396 | 8.138.889 | *** |
|  |  | OF | 3.365.063 | 3.036.667 |  |
|  |  | NF | 2.966.274 | 7.499.167 | *** |
|  |  | RF | 3.144.911 | 4.853.252 | ** |
|  | CO | AF | 3.324.396 | -8.138.889 | *** |
|  |  | OF | 2.659.517 | -5.102.222 | *** |
|  |  | NF | 2.132.642 | -639.722 |  |
|  |  | RF | 2.374.836 | -3.285.637 | ** |
|  | OF | AF | 3.365.063 | -3.036.667 |  |
|  |  | CO | 2.659.517 | 5.102.222 | *** |
|  |  | NF | 2.195.496 | 44.625 | *** |
|  |  | RF | 2.431.437 | 1.816.585 |  |
|  | NF | AF | 2.966.274 | -7.499.167 | *** |
|  |  | CO | 2.132.642 | 639.722 |  |
|  |  | OF | 2.195.496 | -44.625 | *** |
|  |  | RF | 1.840.385 | -2.645.915 | ** |
|  | RF | AF | 3.144.911 | -4.853.252 | ** |
|  |  | CO | 2.374.836 | 3.285.637 | ** |
|  |  | OF | 2.431.437 | -1.816.585 |  |
|  |  | NF | 1.840.385 | 2.645.915 | ** |
| bio_16 | AF | CO | 3.546.288 | -3.757.407 | * |
|  |  | OF | 3.589.669 | -615.333 |  |
|  |  | NF | 3.164.263 | -4.396.458 | ** |
|  |  | RF | 3.354.823 | -3.612.602 | * |
|  | CO | AF | 3.546.288 | 3.757.407 | * |
|  |  | OF | 283.703 | 3.142.074 | * |
|  |  | NF | 2.274.989 | -639.051 |  |
|  |  | RF | 2.533.348 | 144.806 |  |
|  | OF | AF | 3.589.669 | 615.333 |  |
|  |  | CO | 283.703 | -3.142.074 | * |
|  |  | NF | 2.342.038 | -3.781.125 | ** |
|  |  | RF | 2.593.727 | -2.997.268 | * |
|  | NF | AF | 3.164.263 | 4.396.458 | ** |
|  |  | CO | 2.274.989 | 639.051 |  |
|  |  | OF | 2.342.038 | 3.781.125 | ** |
|  |  | RF | 1.963.225 | 783.857 |  |
|  | RF | AF | 3.354.823 | 3.612.602 | * |
|  |  | CO | 2.533.348 | -144.806 |  |
|  |  | OF | 2.593.727 | 2.997.268 | * |
|  |  | NF | 1.963.225 | -783.857 |  |
| bio_17 | AF | CO | 3.162.391 | 1.676.852 |  |
|  |  | OF | 3.201.076 | -6.073.667 | *** |
|  |  | NF | 2.821.721 | -5.699.167 | *** |
|  |  | RF | 2.991.653 | -4.968.496 | ** |
|  | CO | AF | 3.162.391 | -1.676.852 |  |
|  |  | OF | 2.529.913 | -7.750.519 | *** |
|  |  | NF | 2.028.714 | -7.376.019 | *** |
|  |  | RF | 2.259.105 | -6.645.348 | *** |
|  | OF | AF | 3.201.076 | 6.073.667 | *** |
|  |  | CO | 2.529.913 | 7.750.519 | *** |
|  |  | NF | 2.088.505 | 3.745 |  |
|  |  | RF | 2.312.948 | 1.105.171 |  |
|  | NF | AF | 2.821.721 | 5.699.167 | *** |
|  |  | CO | 2.028.714 | 7.376.019 | *** |
|  |  | OF | 2.088.505 | -3.745 |  |
|  |  | RF | 1.750.699 | 730.671 |  |
|  | RF | AF | 2.991.653 | 4.968.496 | ** |
|  |  | CO | 2.259.105 | 6.645.348 | *** |
|  |  | OF | 2.312.948 | -1.105.171 |  |
|  |  | NF | 1.750.699 | -730.671 |  |
| bio_18 | AF | CO | 3.246.985 | -38.125 | * |
|  |  | OF | 3.286.705 | -4.661.167 | ** |
|  |  | NF | 2.897.202 | -8.482.292 | *** |
|  |  | RF | 3.071.679 | -4.173.069 | ** |
|  | CO | AF | 3.246.985 | 38.125 | * |
|  |  | OF | 2.597.588 | -848.667 |  |
|  |  | NF | 2.082.982 | -4.669.792 | *** |
|  |  | RF | 2.319.536 | -360.569 |  |
|  | OF | AF | 3.286.705 | 4.661.167 | ** |
|  |  | CO | 2.597.588 | 848.667 |  |
|  |  | NF | 2.144.372 | -3.821.125 | *** |
|  |  | RF | 2.374.819 | 488.098 |  |
|  | NF | AF | 2.897.202 | 8.482.292 | *** |
|  |  | CO | 2.082.982 | 4.669.792 | *** |
|  |  | OF | 2.144.372 | 3.821.125 | *** |
|  |  | RF | 179.753 | 4.309.223 | *** |
|  | RF | AF | 3.071.679 | 4.173.069 | ** |
|  |  | CO | 2.319.536 | 360.569 |  |
|  |  | OF | 2.374.819 | -488.098 |  |
|  |  | NF | 179.753 | -4.309.223 | *** |
| bio_19 | AF | CO | 3.465.914 | 7.248.148 | *** |
|  |  | OF | 3.508.312 | 2.803.333 |  |
|  |  | NF | 3.092.547 | 3.322.083 | * |
|  |  | RF | 3.278.788 | 5.685.772 | *** |
|  | CO | AF | 3.465.914 | -7.248.148 | *** |
|  |  | OF | 2.772.731 | -4.444.815 | ** |
|  |  | NF | 2.223.428 | -3.926.065 | *** |
|  |  | RF | 2.475.932 | -1.562.376 |  |
|  | OF | AF | 3.508.312 | -2.803.333 |  |
|  |  | CO | 2.772.731 | 4.444.815 | ** |
|  |  | NF | 2.288.957 | 51.875 |  |
|  |  | RF | 2.534.942 | 2.882.439 | * |
|  | NF | AF | 3.092.547 | -3.322.083 | * |
|  |  | CO | 2.223.428 | 3.926.065 | *** |
|  |  | OF | 2.288.957 | -51.875 |  |
|  |  | RF | 191.873 | 2.363.689 | * |
|  | RF | AF | 3.278.788 | -5.685.772 | *** |
|  |  | CO | 2.475.932 | 1.562.376 |  |
|  |  | OF | 2.534.942 | -2.882.439 | * |
|  |  | NF | 191.873 | -2.363.689 | * |
| distance | AF | CO | 2.732.319 | -14.875.463 | *** |
|  |  | OF | 2.765.743 | -4.539.167 | ** |
|  |  | NF | 2.437.978 | -7.274.792 | *** |
|  |  | RF | 25.848 | -8.793.801 | *** |
|  | CO | AF | 2.732.319 | 14.875.463 | *** |
|  |  | OF | 2.185.855 | 10.336.296 | *** |
|  |  | NF | 1.752.817 | 7.600.671 | *** |
|  |  | RF | 1.951.876 | 6.081.662 | *** |
|  | OF | AF | 2.765.743 | 4.539.167 | ** |
|  |  | CO | 2.185.855 | -10.336.296 | *** |
|  |  | NF | 1.804.476 | -2.735.625 | ** |
|  |  | RF | 1.998.396 | -4.254.634 | *** |
|  | NF | AF | 2.437.978 | 7.274.792 | *** |
|  |  | CO | 1.752.817 | -7.600.671 | *** |
|  |  | OF | 1.804.476 | 2.735.625 | ** |
|  |  | RF | 1.512.611 | -1.519.009 | * |
|  | RF | AF | 25.848 | 8.793.801 | *** |
|  |  | CO | 1.951.876 | -6.081.662 | *** |
|  |  | OF | 1.998.396 | 4.254.634 | *** |
|  |  | NF | 1.512.611 | 1.519.009 | * |
| eastness | AF | CO | 3.564.113 | -3.593.519 | * |
|  |  | OF | 3.607.712 | -6.220.333 | *** |
|  |  | NF | 3.180.167 | -4.573.333 | ** |
|  |  | RF | 3.371.685 | -5.710.772 | ** |
|  | CO | AF | 3.564.113 | 3.593.519 | * |
|  |  | OF | 285.129 | -2.626.815 |  |
|  |  | NF | 2.286.423 | -979.815 |  |
|  |  | RF | 2.546.081 | -2.117.254 |  |
|  | OF | AF | 3.607.712 | 6.220.333 | *** |
|  |  | CO | 285.129 | 2.626.815 |  |
|  |  | NF | 2.353.809 | 16.47 |  |
|  |  | RF | 2.606.764 | 509.561 |  |
|  | NF | AF | 3.180.167 | 4.573.333 | ** |
|  |  | CO | 2.286.423 | 979.815 |  |
|  |  | OF | 2.353.809 | -16.47 |  |
|  |  | RF | 1.973.092 | -1.137.439 |  |
|  | RF | AF | 3.371.685 | 5.710.772 | ** |
|  |  | CO | 2.546.081 | 2.117.254 |  |
|  |  | OF | 2.606.764 | -509.561 |  |
|  |  | NF | 1.973.092 | 1.137.439 |  |
| geology | AF | CO | 3.023.192 | 11.892.593 | *** |
|  |  | OF | 3.060.174 | 36.52 | * |
|  |  | NF | 2.697.517 | 91.9 | *** |
|  |  | RF | 2.859.969 | 7.656.098 | *** |
|  | CO | AF | 3.023.192 | -11.892.593 | *** |
|  |  | OF | 2.418.554 | -8.240.593 | *** |
|  |  | NF | 1.939.416 | -2.702.593 | ** |
|  |  | RF | 2.159.666 | -4.236.495 | *** |
|  | OF | AF | 3.060.174 | -36.52 | * |
|  |  | CO | 2.418.554 | 8.240.593 | *** |
|  |  | NF | 1.996.575 | 55.38 | *** |
|  |  | RF | 2.211.139 | 4.004.098 | *** |
|  | NF | AF | 2.697.517 | -91.9 | *** |
|  |  | CO | 1.939.416 | 2.702.593 | ** |
|  |  | OF | 1.996.575 | -55.38 | *** |
|  |  | RF | 1.673.639 | -1.533.902 |  |
|  | RF | AF | 2.859.969 | -7.656.098 | *** |
|  |  | CO | 2.159.666 | 4.236.495 | *** |
|  |  | OF | 2.211.139 | -4.004.098 | *** |
|  |  | NF | 1.673.639 | 1.533.902 |  |
| northness | AF | CO | 3.523.925 | -1.362.963 |  |
|  |  | OF | 3.567.033 | -6.170.667 | *** |
|  |  | NF | 3.144.309 | -3.249.167 | * |
|  |  | RF | 3.333.668 | -4.642.276 | ** |
|  | CO | AF | 3.523.925 | 1.362.963 |  |
|  |  | OF | 281.914 | -4.807.704 | *** |
|  |  | NF | 2.260.643 | -1.886.204 |  |
|  |  | RF | 2.517.373 | -3.279.313 | * |
|  | OF | AF | 3.567.033 | 6.170.667 | *** |
|  |  | CO | 281.914 | 4.807.704 | *** |
|  |  | NF | 2.327.269 | 29.215 | * |
|  |  | RF | 2.577.371 | 152.839 |  |
|  | NF | AF | 3.144.309 | 3.249.167 | * |
|  |  | CO | 2.260.643 | 1.886.204 |  |
|  |  | OF | 2.327.269 | -29.215 | * |
|  |  | RF | 1.950.845 | -139.311 |  |
|  | RF | AF | 3.333.668 | 4.642.276 | ** |
|  |  | CO | 2.517.373 | 3.279.313 | * |
|  |  | OF | 2.577.371 | -152.839 |  |
|  |  | NF | 1.950.845 | 139.311 |  |
| CTI | AF | CO | 3.381.421 | -1.177.778 |  |
|  |  | OF | 3.422.785 | -69.68 | *** |
|  |  | NF | 3.017.156 | -4.894.375 | ** |
|  |  | RF | 3.198.857 | -6.632.927 | *** |
|  | CO | AF | 3.381.421 | 1.177.778 |  |
|  |  | OF | 2.705.137 | -5.790.222 | *** |
|  |  | NF | 2.169.224 | -3.716.597 | *** |
|  |  | RF | 2.415.572 | -5.455.149 | *** |
|  | OF | AF | 3.422.785 | 69.68 | *** |
|  |  | CO | 2.705.137 | 5.790.222 | *** |
|  |  | NF | 2.233.156 | 2.073.625 |  |
|  |  | RF | 2.473.144 | 335.073 |  |
|  | NF | AF | 3.017.156 | 4.894.375 | ** |
|  |  | CO | 2.169.224 | 3.716.597 | *** |
|  |  | OF | 2.233.156 | -2.073.625 |  |
|  |  | RF | 1.871.954 | -1.738.552 |  |
|  | RF | AF | 3.198.857 | 6.632.927 | *** |
|  |  | CO | 2.415.572 | 5.455.149 | *** |
|  |  | OF | 2.473.144 | -335.073 |  |
|  |  | NF | 1.871.954 | 1.738.552 |  |
